# Research trend of functional magnetic resonance imaging in diabetes mellitus research: a visualization and bibliometric analysis

**DOI:** 10.3389/fneur.2025.1539995

**Published:** 2025-08-21

**Authors:** Zongbo Sun, Yuanyuan Li, Xueli Qu, Luguang Wang, Shengyin Zhu, Xuejing Sun, Li Yang, Xiaonan Sun

**Affiliations:** ^1^School of Medicine, Liao Cheng University, Liaocheng, Shandong, China; ^2^Department of Radiology, Dongchangfu District Maternal and Child Health Hospital, Liaocheng, Shandong, China; ^3^Department of Radiology, Dongchangfu District People’s Hospital, Liaocheng, Shandong, China; ^4^Medical Genetics Laboratory, Dongchangfu District Maternal and Child Health Hospital, Liaocheng, Shandong, China

**Keywords:** diabetes mellitus, bibliometrics, VOSviewer, CiteSpace, functional magnetic resonance imaging

## Abstract

**Background:**

Understanding the neurological complications associated with diabetes mellitus is essential for developing comprehensive treatment strategies. Functional magnetic resonance imaging (fMRI) is a powerful tool for investigating brain functional and structural changes associated with various conditions, including diabetes mellitus.

**Objectives:**

To analyze the application trends, research hotspots, and emerging frontiers of fMRI in diabetes mellitus research through a comprehensive bibliometric analysis.

**Methods:**

A systematic literature search was conducted utilizing the Web of Science Core Collection (WoSCC) database. Bibliometric tools, including VOSviewer (version 1.6.20), CiteSpace (version 6.3.R1), and R (version 4.3.3), were employed for data analysis.

**Results:**

A total of 706 articles about fMRI and diabetes mellitus were published from 1987 to 2024. The United States of America (USA) ranks first (*n* = 931), followed by China (*n* = 756) and Germany (*n* = 270) regarding total publications. Harvard University ranks first in terms of total publications. Among the top ten institutions regarding publications, the majority of articles originated from the USA. The journal *Diabetes* has the highest number of publications. The author SHAO YI ranks first in total publications, while FRITSCHE ANDREAS ranks first in total citations. The top five keywords identified are “dementia,” “risk,” “brain,” “Alzheimer’s disease,” and “functional connectivity.” Keyword burst analysis indicates that the recent research hotspots included “impairment,” “dysfunction,” and “diagnosis.”

**Conclusion:**

Cognitive impairment and dysfunction related to diabetes mellitus, along with Alzheimer’s disease and dementia, and their diagnosis were identified as focal areas of research. Future investigations should concentrate on predicting and early diagnosing cognitive function in patients with diabetes mellitus using fMRI. The findings of this study provide a valuable reference for researchers and clinicians seeking to explore the neurological dimensions of diabetes mellitus and develop targeted therapeutic approaches.

## 1 Introduction

Diabetes mellitus is a global health concern affecting millions of individuals worldwide, with its prevalence steadily increasing (1). Diabetes mellitus is a chronic disease associated with complications that significantly impact patients’ quality of life and healthcare systems (2). Among these complications, neurological issues, particularly cognitive impairment and structural brain abnormalities (3, 4), have gained considerable attention in recent years. Cognitive impairment mainly includes Alzheimer’s disease (AD) and vascular dementia. People with type 2 diabetes mellitus (T2DM) have twice the risk of mild cognitive impairment and dementia as non-diabetics, with about 20% of those over 60 years of age at risk of progression to dementia (5). The diagnosis of diabetes mellitus-related cognitive dysfunction mainly relies on neuropsychological scales (6, 7), which may result in misdiagnosis. Besides, diabetic peripheral neuropathy (DPN) is one of the most common chronic complications of diabetes mellitus, with a prevalence of 50% (8, 9). The development of foot ulcers is a serious consequence of the progression of DPN, possibly leading to amputation and death (9). Therefore, early diagnosis and treatment are particularly important. The current gold standard method for diagnosing DPN is electromyography (EMG). Although many patients with DPN show evidence of neurologic deficits on EMG, they do not present with neuropathic symptoms. Due to its insidious onset, DPN often goes undiagnosed in its early stages (10).

Functional magnetic resonance imaging (fMRI) has become a crucial tool in neuroscience, allowing researchers to non-invasively study brain activity and structural changes (11–13). In the context of diabetes mellitus, fMRI has been employed to explore the neurological impacts of the disease, revealing critical insights into cognitive function, brain connectivity, and neurovascular changes (14, 15). fMRI can clearly show the anatomical structure of brain tissue and neuropathological features (16), which is important for diagnosing and treating T2DM cognitive dysfunction. Research has shown that patients with T2DM cognitive dysfunction have abnormal structural changes on fMRI (17). Furthermore, fMRI has become a practical tool for DPN imaging because of its noninvasive, radiation-free characteristics and its ability to reflect in real time the local oxygen consumption of various regions of the CNS in response to external stimuli (10, 18). Although some reviews have discussed the fMRI study of diabetes mellitus, no research has summarized the overview, hotspots, and trends of the application of fMRI in diabetes mellitus using visualization and analysis methods.

Bibliometrics is the cross-cutting science that quantitatively analyzes all knowledge carriers using mathematical and statistical methods. Its main measurement objects are the amount of literature, the number of authors, and vocabulary. The amount of literature is mainly the variety of publications and journals and where lies the majority of the literature. The number of authors mainly refers to the researcher’s personal or group. Vocabulary is mainly the variety of literature markers used to classify most papers (19). Bibliometrics research methods have been applied in many research areas, in medicine and other fields. Currently, literature research is often used in clinical settings to summarize the research frontiers and trends of certain diseases and to provide directions for clinical disease research (20).

Therefore, this study aimed to perform a bibliometric analysis of the application of fMRI in diabetes mellitus research. By systematically analyzing publication trends, research hotspots, and collaboration networks, this study sought to map the current state of research, identify key focus areas, and highlight emerging trends. The findings will provide valuable insights for researchers and clinicians aiming to further explore and address the neurological complications of diabetes mellitus using fMRI.

## 2 Methods

### 2.1 Search strategies and data collection

We conducted a literature search on the Web of Science Core Collection (WoSCC) on November 12, 2024. The search queries were used to retrieve articles about fMRI and diabetes mellitus published between 1987 and 2024: Searched Method: TS = (functional magnetic resonance imaging* OR “functional MRI*” OR fMRI* OR (“functional” NEAR/3 “magnetic resonance”) OR ((“functional” NEAR/3 “imag*”) AND (magnetic OR resonance OR MR))) AND TS = (diabet* OR mellitus OR T1DM OR T2DM OR hyperglycemia OR glucose intolerance). All information was collected in plain text file format. The following basic information was collected for each article: titles, author information, institutions, countries/regions, keywords, journals, and references. Articles that met the following criteria were included: (1) published in English, and (2) articles on fMRI and diabetes mellitus, including original research. The exclusion criteria were (1) not related to fMRI in diabetes mellitus, (2) non-article document type (e.g., review, case report, letter, and conference abstracts), (3) duplicated publications, or (4) non-English language.

### 2.2 Statistical analysis

Relevant data were extracted from the retrieved literature bibliographies, and used Microsoft Excel to identify and calculate the bibliometric indicators. These indicators cover key aspects of publications, including annual publication counts, citation frequencies, average citation rates, journal names, journal impact factors, publication countries/regions, publishing institutions, and authors. Excel allows researchers to organize and analyze bibliometric data efficiently. During the visual analysis process, three powerful bibliometric analysis tools were used to analyze the academic data comprehensively. These tools are VOSviewer (version 1.6.20), CiteSpace (version 6.3.R1), and R 4.3.3.^1^ VOSviewer is a versatile software tool that plays a crucial role in mapping institutional collaboration, author collaboration, co-authorship, citations, and co-citations (21). VOSviewer allowed the visualization and exploration of complex collaboration and relationship networks within the academic domain, gaining deeper insights into the interconnections among authors, institutions, and publications. VOSviewer was used for keyword co-occurrence analysis, and CiteSpace software was used for keyword burst detection to delve further into emerging trends and research hotspots within our study field. The study employed CiteSpace 6.1.R1 for keyword co-occurrence analysis, setting parameters for time slicing from January 1981 to November 2024 (the initial publications in this field appeared in 1981). The time slice was set to 1 year; node types: keywords. When the node type was keywords, the threshold (top N in each slice) was 5, and the pruning method was pathfinder + pruning merged networks. Based on the parameter settings of each node, visual analysis was conducted to generate a keyword timeline map for the field of “task-based functional magnetic resonance imaging” research. The size of the nodes represents the number of publications, the thickness of the lines represents the strength of the link, and the color of the nodes stands for different clusters or times. The H-index was employed to quantify the academic impact of individuals and journals, respectively. The H-index is a vital indicator for evaluating the academic contribution of researchers and could predict their future scientific achievements (22, 23). In this study, the H-index of each author was obtained from WoSCC. The G-index refers to the highest number of papers that receive H-index or more citations (24). The M-index, defined as (h-index)/(number of years since the author’s first published paper), characterizes the rise in the H-index over time.

## 3 Results

### 3.1 Publication outputs and trends

**Figure 1:**
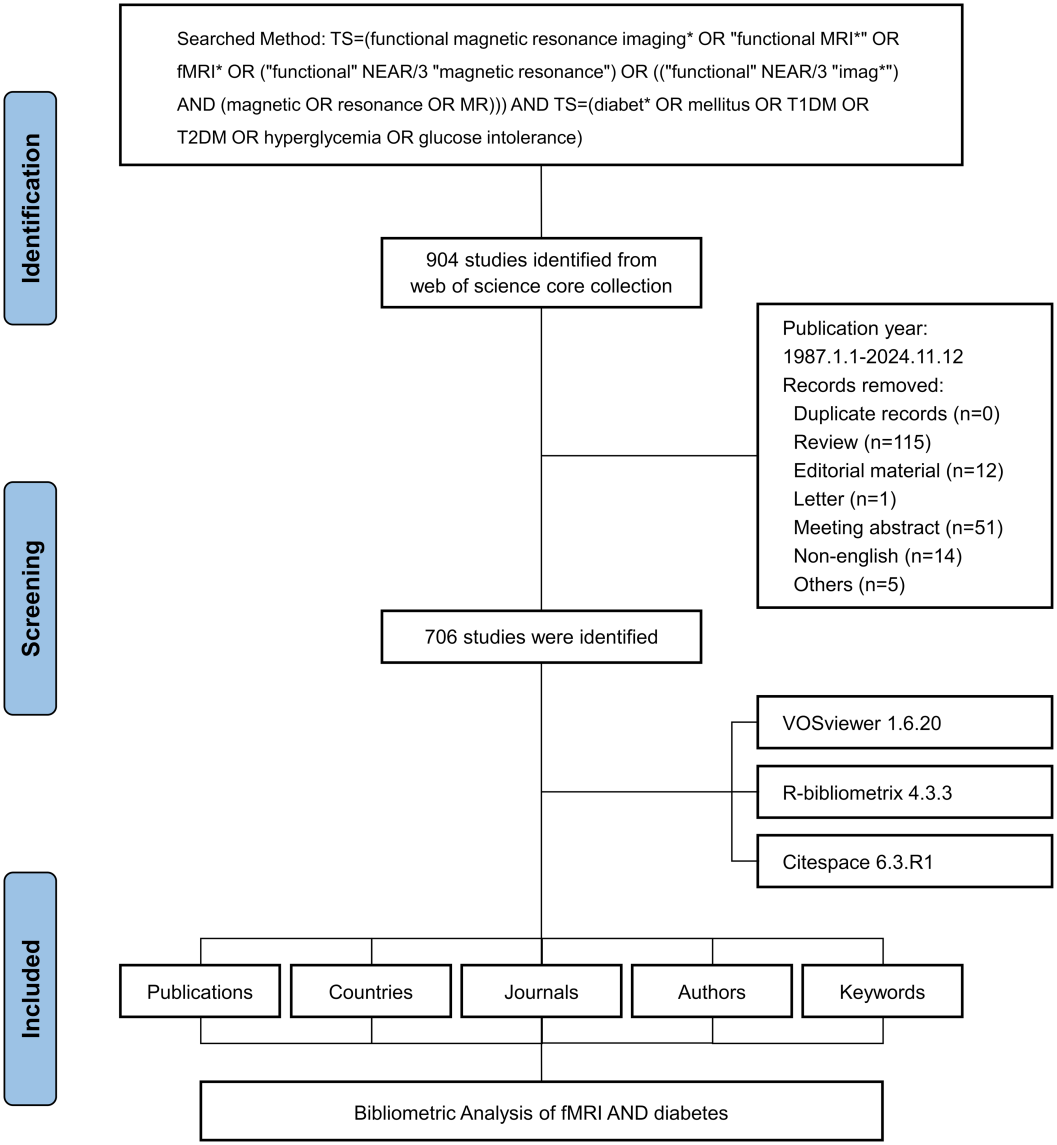
Flowchart of the literature screening process.

**Figure 2:**
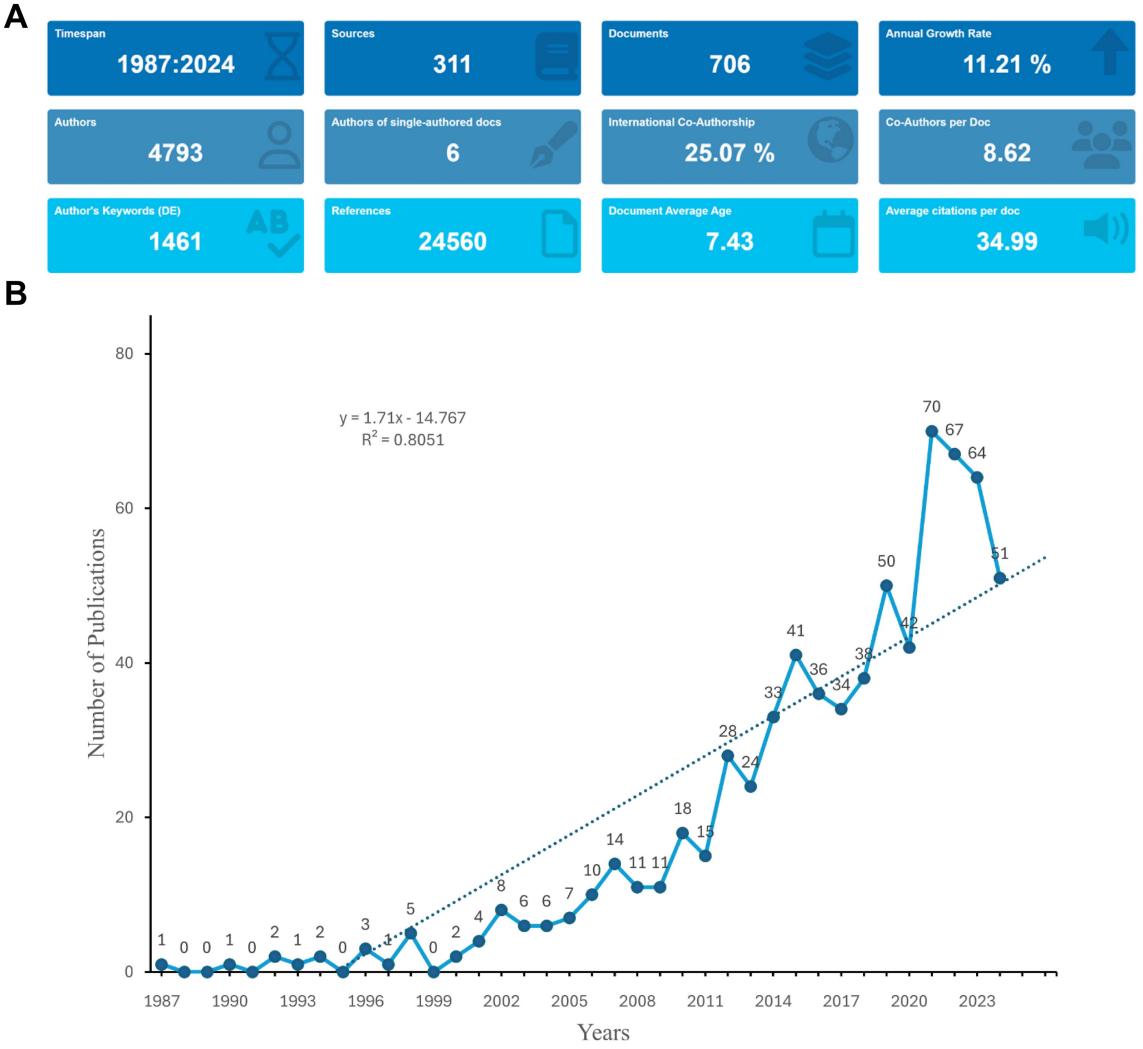
Overall analysis. **(A)** Summary information of the included studies. **(B)** The specific number of annual publications regarding diabetes mellitus and fMRI from 1987 to 2024.

In total, 706 eligible publications were analyzed in the present study. The flowchart of data screening is shown in Figure 1. The investigation showed that 4,793 authors contributed to producing 706 manuscripts in this study. These works were published in 311 journals from 1987 to 2024, citing 24,560 references (Figure 2A). Figure 2B presents the specific number of annual publications regarding the application of fMRI in diabetes mellitus. Since 2006, fMRI and diabetes mellitus research has shown an upward trend. The annual publication volume exceeds 10 articles. The greatest number of annual publications was 70 in 2021. In addition, the index function y = 1.71x-14.767 (R^2^ = 0.8051) of the annual publication trend was determined to evaluate the changing trend between fMRI and diabetes mellitus studies.

### 3.2 Analysis of countries/regions

**Figure 3:**
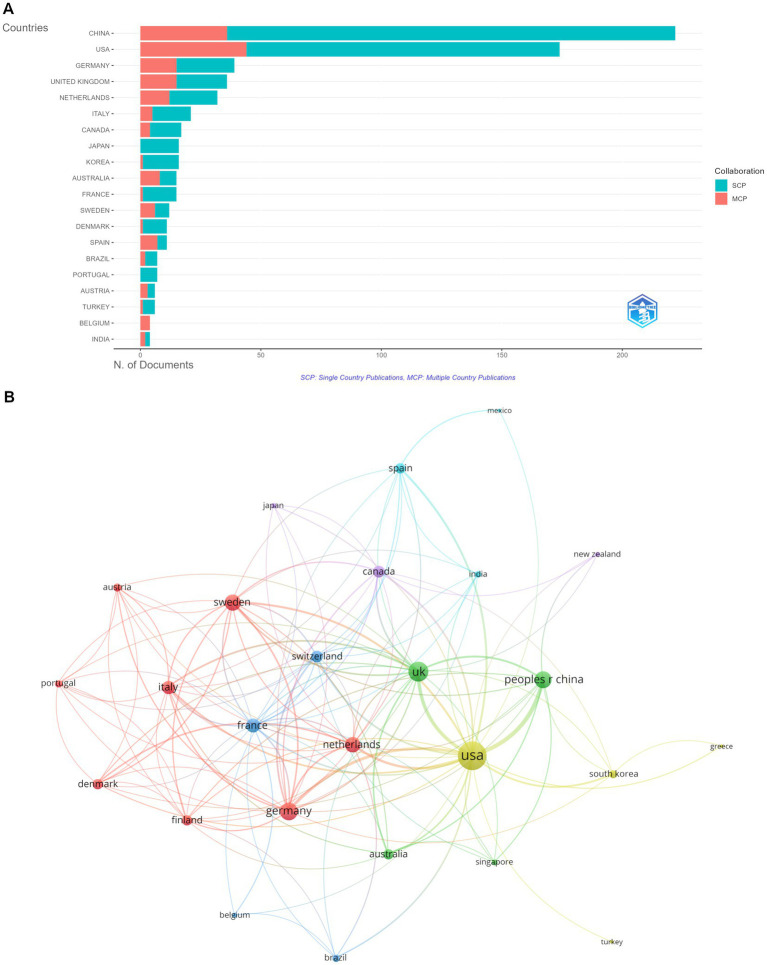
Analysis of countries. **(A)** Distribution of corresponding author’s publications by country. **(B)** Visualization map depicting the collaboration among different countries.

Table 1 presents the top 20 countries/regions based on the number of publications. The United States of America (USA) ranks first (*n* = 931) regarding total publication (TP), followed by China (*n* = 756) and Germany (*n* = 270). The USA also has the leading position (*n* = 10,258) in total citations (TCs), followed by China (*n* = 3,299) and Netherlands (*n* = 2086). Besides, the majority of the articles are single-country publications (SCPs) (Figure 3A). For example, more than 180 articles are SCPs in China. In the USA, 130 articles are SCPs. Among the 26 countries involved in international collaborations with a minimum of three articles, the USA has the highest number of collaborations with other countries (*n* = 151), followed by the United Kingdom (*n* = 70) and Germany (*n* = 59) (Figure 3B).

**Table 1:** Publication and citation profiles of leading countries.

Country	Articles	Freq	SCP	MCP	MCP_Ratio	TP	TP_rank	TC	TC_rank	Average citations
China	222	0.314	186	36	0.162	756	2	3,299	2	14.9
United States of America	174	0.246	130	44	0.253	931	1	10,258	1	59
Germany	39	0.055	24	15	0.385	270	3	1975	4	50.6
United Kingdom	36	0.051	21	15	0.417	195	4	924	6	25.7
Netherlands	32	0.045	20	12	0.375	146	5	2086	3	65.2
Italy	21	0.030	16	5	0.238	108	6	853	7	40.6
Canada	17	0.024	13	4	0.235	98	7	719	8	42.3
Japan	16	0.023	16	0	0.000	66	11	534	9	33.4
Korea	16	0.023	15	1	0.063	78	10	195	13	12.2
Australia	15	0.021	7	8	0.533	61	12	1,458	5	97.2
France	15	0.021	14	1	0.067	97	8	507	10	33.8
Sweden	12	0.017	6	6	0.500	81	9	261	11	21.8
Denmark	11	0.016	10	1	0.091	55	13	85	18	7.7
Spain	11	0.016	4	7	0.636	45	15	236	12	21.5
Brazil	7	0.010	5	2	0.286	30	17	184	14	26.3
Portugal	7	0.010	7	0	0.000	45	14	65	20	9.3
Austria	6	0.008	3	3	0.500	31	16	134	16	22.3
Turkey	6	0.008	5	1	0.167	12	23	145	15	24.2
Belgium	4	0.006	0	4	1.000	10	24	40	23	10
India	4	0.006	2	2	0.500	10	25	62	21	15.5

Articles: Publications of Corresponding Authors only. TP, total publications; TP _rank, rank of total publications; TC, total citations; TC _rank, rank of total citations; Average Citations, the average number of citations per publication.

### 3.3 Analysis of institutions

**Figure 4:**
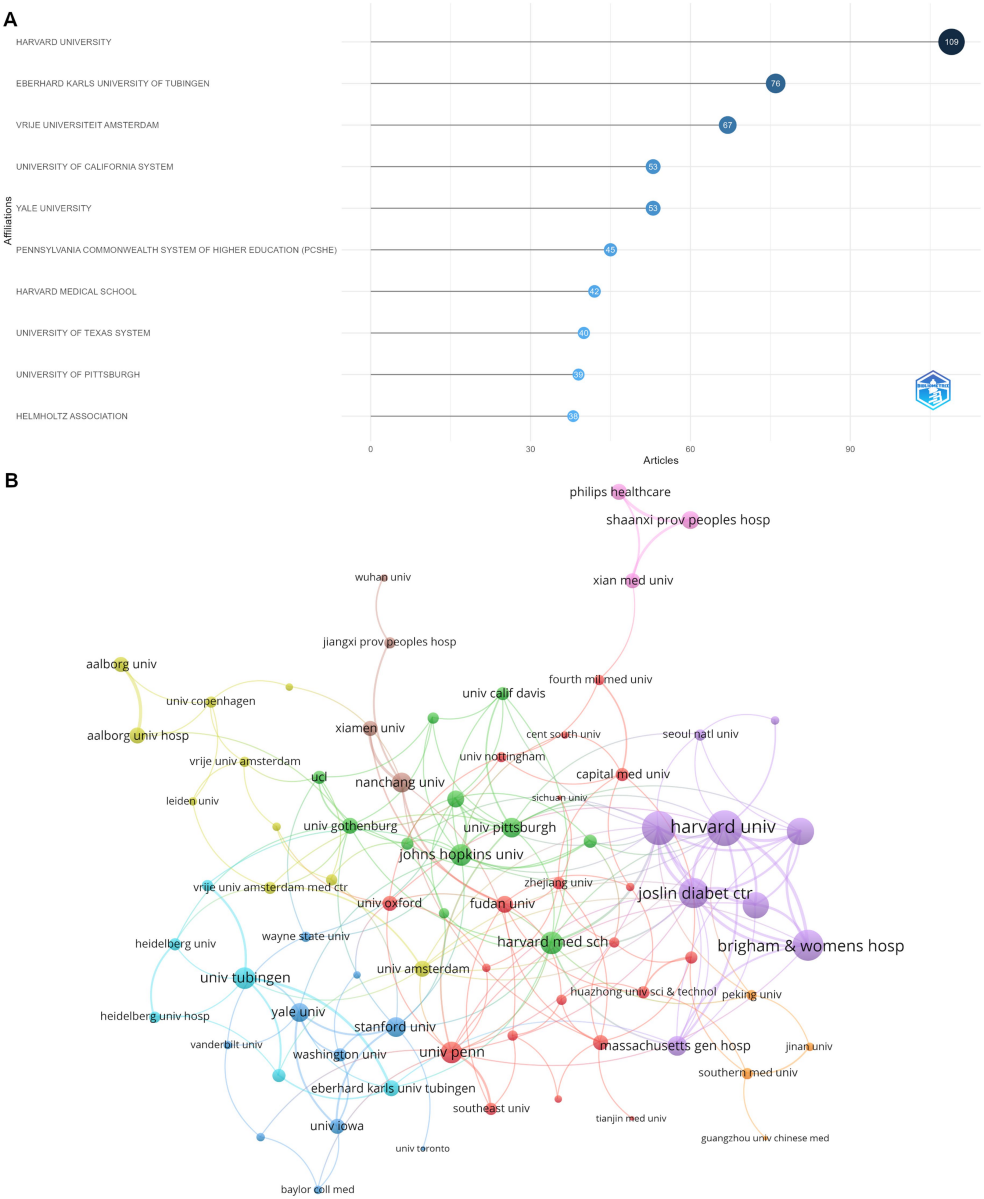
Analysis of institutions. **(A)** Top 10 institutions according to the publications and citations. **(B)** Visualization map depicting the collaboration among different institutions.

Figure 4A reports the top ten institutions by article count and rank. Harvard University in the USA ranks first with 109 publications, and Eberhard Karls University of Tubingen in Germany ranks second with 76 publications. Figure 4B depicts the institutional collaboration network map. Among the 77 institutions involved in international collaborations with a minimum of five articles, Harvard University has the highest number of collaborations with other countries (total link strength = 46), followed by Beth Israel Deaconess Medical Center (total link strength = 42) and Brigham and Women’s Hospital (total link strength = 34).

### 3.4 Analysis of journals

Table 2 shows the basic information of the top 20 most productive journals in fMRI and diabetes mellitus. The journal with the highest impact factor (IF) was *Diabetes Care* (IF = 14.8), followed by *Cardiovascular Diabetology* (IF = 8.5). *Diabetes* has published the greatest TP of 26 publications, followed by *Frontiers in Neuroscience* (23 publications) and *Diabetes Care* (20 publications). In addition, 95% of the top 20 journals were categorized in the Q1 or Q2 Journal Citation Reports (JCR) region. Regarding citations, articles published in *Neuroimage* were cited most frequently (1,314 times), followed by *Diabetes* (1,053 times). Regarding H-index, *Diabetes Care* was in the leading position (H = 22).

**Table 2:** Bibliometric indicators of high-impact journals.

Journal	H_index	IF	total citations _Quartile	TP	TP_rank	TC	TC_rank
Diabetes	22	6.2	Q1	26	1	1,053	2
Diabetes Care	16	14.8	Q1	20	3	1,027	3
PLOS One	11	2.9	Q1	20	4	373	11
Scientific Reports	11	3.8	Q1	13	7	185	27
Frontiers in Neuroscience	10	3.2	Q2	23	2	176	29
Neuroimage	10	4.7	Q1	10	10	1,314	1
Frontiers in Aging Neuroscience	9	4.1	Q2	18	5	156	34
Stroke	9	7.8	Q1	10	11	524	4
Journal of Magnetic Resonance Imaging	8	3.3	Q1	12	8	226	19
Diabetologia	7	8.4	Q1	7	16	445	7
European Radiology	7	4.7	Q1	9	14	129	43
Cardiovascular Diabetology	6	8.5	Q1	9	12	85	68
Human Brain Mapping	6	3.5	Q1	6	18	406	9
Journal of Alzheimer’s Disease	6	3.4	Q2	6	19	190	23
Journal of Clinical Endocrinology & Metabolism	6	5	Q1	8	15	233	18
Neuroimage-Clinical	6	3.4	Q2	6	20	124	48
Diabetes Obesity & Metabolism	5	5.4	Q1	9	13	76	76
Frontiers in Endocrinology	5	3.9	Q2	15	6	36	164
Investigative Ophthalmology & Visual Science	5	5	Q1	5	24	149	37
Brain Research	4	2.7	Q3	4	26	134	41

H-index: The h-index of the journal, which measures both the productivity and citation impact of the publications. IF: Impact Factor, indicating the average number of citations to recent articles published in the journal. JCR: The quartile ranking of the journal in the Journal Citation Reports, indicating the journal’s ranking relative to others in the same field (Q1: top 25%, Q2: 25–50%, Q3: 50–75%, Q4: bottom 25%). TP, total publications; TP_rank, rank of total publications; TC, total citations; TC_rank, rank of total citations; Average Citations, the average number of citations per publication; PY_start, publication year start, indicating the year the journal started publication.

**Figure 5:**
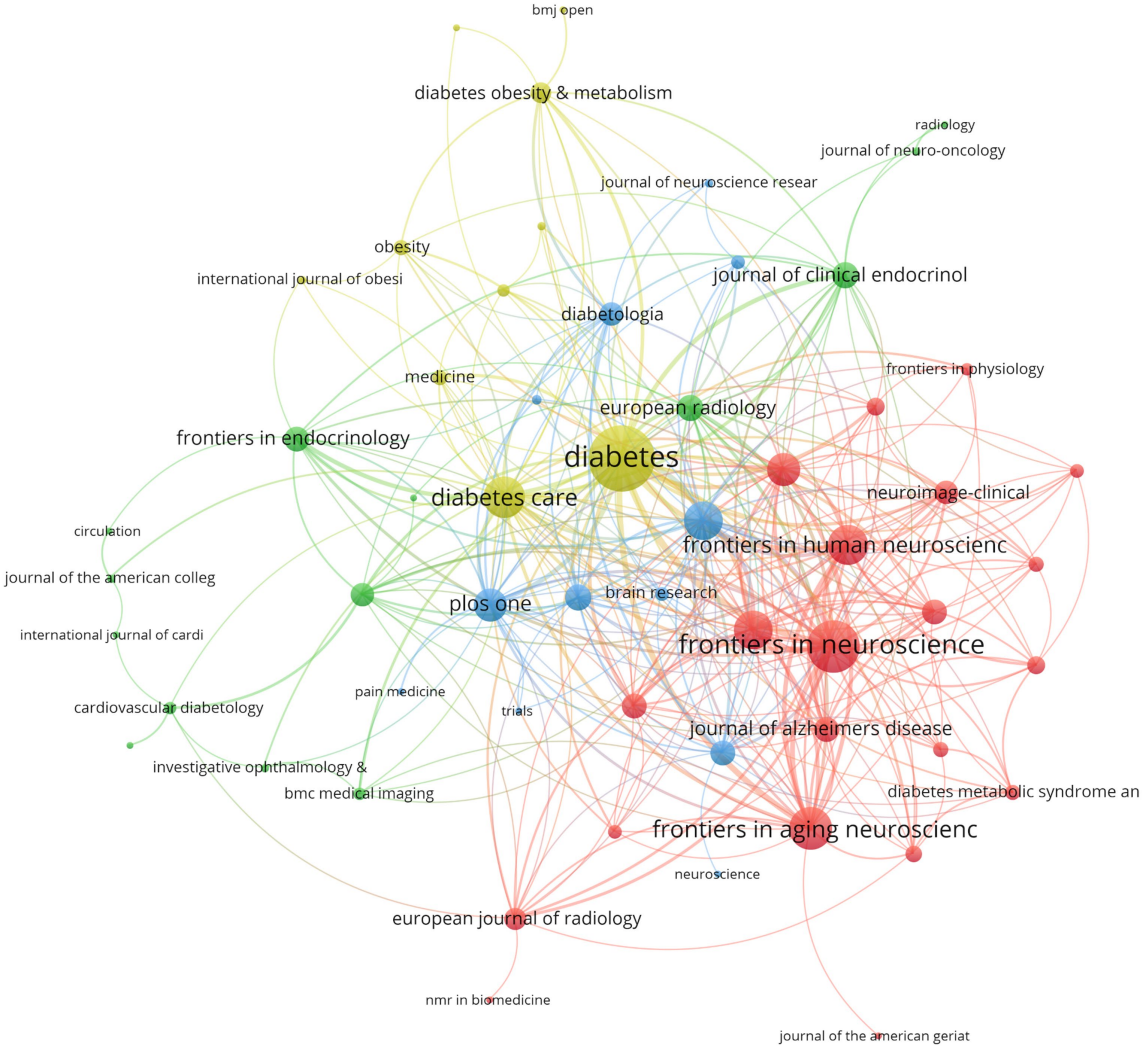
Visualization map depicting the collaboration among different journals.

Figure 5 depicts the network visualization map of the journal co-citation analysis. The co-occurrence networks of journals contained 65 journals with at least three occurrences. The three key journals with the highest total link strength in co-occurrence networks were *Diabetes* (total link strength = 254), *Frontiers in Neuroscience* (total link strength = 164), and *Frontiers in Aging Neuroscience* (total link strength = 109).

### 3.5 Analysis of authors

**Figure 6:**
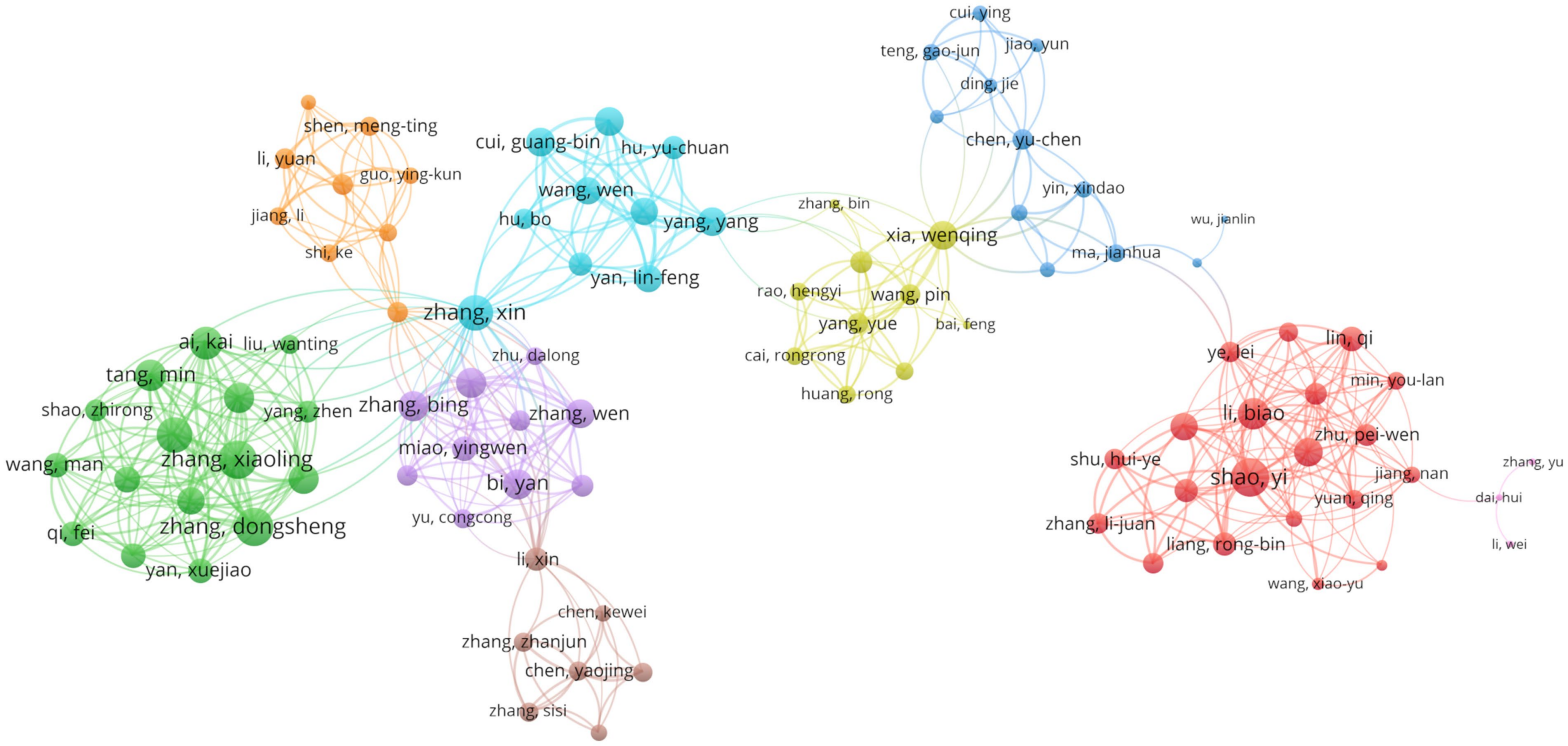
Visualization map depicting the collaboration among different authors.

Table 3 presents the top 20 core authors. The top 20 authors published 210 articles collectively, totaling 9,499 citations. Regarding TP, SHAO YI leads with 16 publications, followed by QIU SHIJUN with 14 publications. Regarding TC, FRITSCHE ANDREAS leads with 962 citations, and PREISSL HUBERT follows with 910 citations. Regarding the H-index and G-index, FRITSCHE ANDREAS is in the leading position. Figure 6 displays the collaborations among authors. Among the 97 authors involved in international collaborations with a minimum of three articles, QIU SHIJUN has the highest number of collaborations with other countries (total link strength = 104), followed by TAN XIN (total link strength = 100) and LIANG YI (total link strength = 85). In the network visualization, the size of the nodes increases with the number of articles published by the authors. The larger the node, the more articles the author publishes.

**Table 3:** Publication and citation profiles of high-impact authors.

Authors	H_index	G-index	M-index	PY_start	TP	TP_Frac	TP_rank	TC	TC_rank
Fritsche Andreas	12	12	0.63	2006	12	1.20	5	962	1
Barkhof Frederik	10	10	0.77	2012	10	1.19	10	648	7
Diamant Michaela	10	10	0.63	2009	10	1.13	12	819	3
Haering Hans-Ulrich	10	10	0.67	2010	10	0.82	13	588	8
Ijzerman Richard G.	10	13	0.77	2012	13	1.75	3	666	6
Preissl Hubert	10	11	0.53	2006	11	1.12	8	910	2
Kullmann Stephanie	9	10	0.60	2010	10	0.83	14	774	4
Heni Martin	8	8	0.62	2012	8	0.64	24	528	9
Shao Yi	8	11	1.33	2019	16	1.68	1	137	33
Veltman Dick J.	8	10	0.73	2014	10	1.44	15	518	10
Xia Wenqing	8	10	0.67	2013	10	1.28	16	323	20
Bolo Nicolas R.	7	8	0.41	2008	8	1.09	22	504	11
Jacobson Alan M.	7	8	0.41	2008	8	1.09	25	504	11
Li Biao	7	8	1.17	2019	8	0.81	26	78	42
Li Yifan	7	11	1.17	2019	11	1.15	7	128	38
Liang Yi	7	11	1.17	2019	12	1.26	6	130	37
Musen Gail	7	8	0.41	2008	8	1.09	27	504	11
Qiu Shijun	7	11	1.17	2019	14	1.41	2	137	33
Simonson Donald C.	7	8	0.41	2008	8	1.09	29	504	11
Tan Xin	7	11	1.17	2019	13	1.31	4	137	33

H-index: The h-index of the journal, which measures both the productivity and citation impact of the publications. TP, total publications; TP_rank, rank of total publications; TC, total citations; TC_rank, rank of total citations; Average Citations, the average number of citations per publication; PY_start, publication year start, indicating the year the author started publication.

### 3.6 Keywords analysis

**Figure 7:**
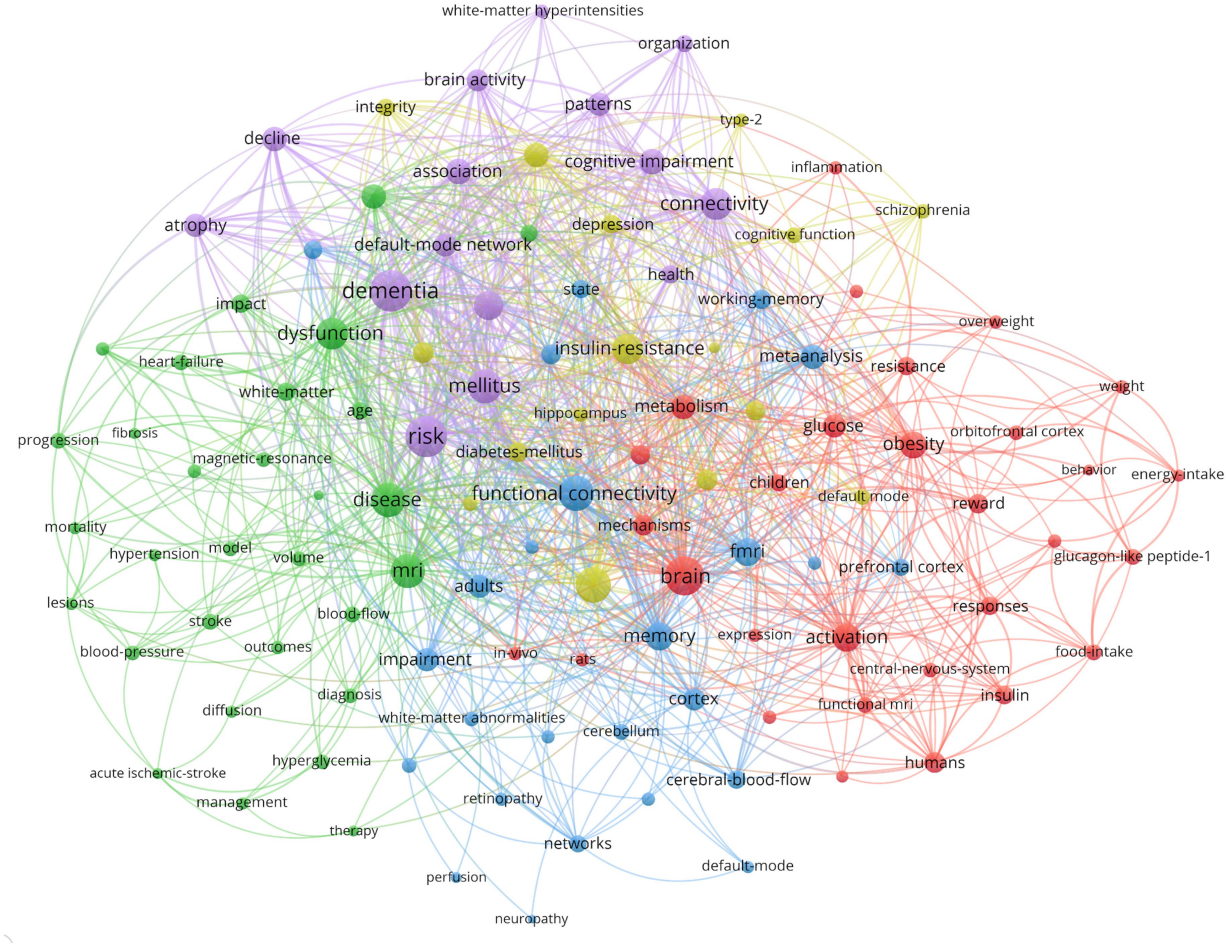
Visual analysis of keyword co-occurrence network analysis.

A comprehensive keyword analysis of the selected articles was performed using “Author Keywords” from the Bibliophagy application and “Keywords Plus” provided by the VOSviewer application. In total, 116 keywords with a minimum of eight occurrences were identified (Figure 7). Table 4 displays the frequency distribution of the keywords in the top 20 most frequent occurrences. Among them, the top five keywords identified were “dementia,” “risk,” “brain,” “Alzheimer’s disease,” and “functional connectivity.”

**Table 4:** Frequency distribution of the top 20 most frequent occurrences keywords.

Keywords	Occurrences	Total link strength
Dementia	64	305
Risk	70	303
Brain	70	268
Alzheimer’s-disease	50	234
Functional connectivity	51	227
Disease	67	217
Mellitus	50	210
MRI	61	209
Dysfunction	44	184
Connectivity	41	177
Insulin-resistance	43	174
Mild cognitive impairment	33	156
fMRI	41	148
Activation	40	147
Memory	31	145
Obesity	31	131
Association	32	125
Cognitive impairment	23	119
Metabolism	29	115
Meta-analysis	27	113

**Figure 8:**
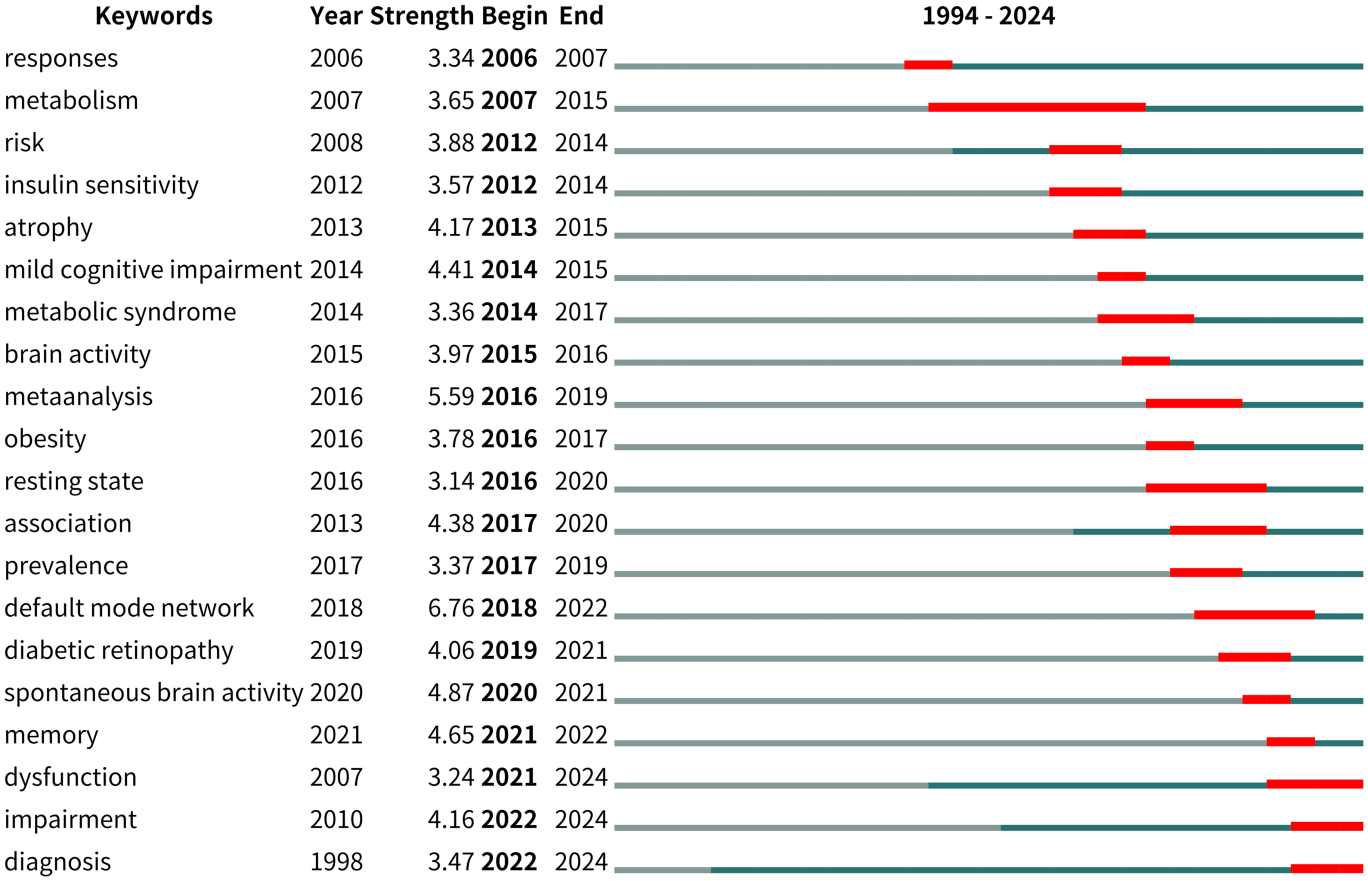
Top 20 keywords with the strongest citation bursts.

In addition, the keyword burst detection was used to identify research hot spots. Figure 8 depicts the top 20 references with the strongest keyword bursts on research during the period of 1994–2024. The green line indicates the 1994–2024 timeframe, and the red line indicates the period during which the outbreak was sustained. Since 2017, the keywords “association,” “prevalence,” “default mode network,” “diabetic retinopathy,” “spontaneous brain activity,” “memory,” “dysfunction,” “impairment,” and “diagnosis” have been more prominently concentrated, indicating promising developments. Among the identified keyword bursts, those featured by the end of 2024 were “dysfunction,” “impairment,” and “diagnosis.”

## 4 Discussion

### 4.1 General information

The present study identified 706 articles about fMRI and diabetes mellitus published from 1987 to 2024. The overall publication numbers maintained a stable growth trend. The USA published the largest number of papers. Among the top ten institutions, the majority are from the USA, suggesting that institutions from the USA lead an important position in this field. *Diabetes* is the journal with the most publications. SHAO YI has the highest publication among all authors.

In terms of journals, *Diabetes* has the highest number of publications in fMRI and diabetes mellitus, followed by *Frontiers in Neuroscience* and *Diabetes Care*. A previous study published in *Diabetes* suggested that cognitive functioning among T1DM can be assessed with fMRI techniques (25). Other studies published in *Frontiers in Neuroscience* used fMRI to acquire brain change data (26). Among the top 20 most cited journals, *Neuroimage* has the most TCs. The journal *Diabetes* is ranked second in the number of papers published. Regarding the H-index, the journal *Diabetes* holds the leading position. In addition, *Neuroimage* and *Diabetes* are both from the USA, demonstrating that the USA provides an important platform for developing fMRI and diabetes mellitus.

Regarding publication volume, the most prolific author is SHAO YI from China. His research focuses on brain activity in patients with diabetic optic neuropathy (27, 28). QIU SHIJUN from China mainly studied the functional connectivity (FC) of patients with T2DM and mild cognitive impairment (29). However, regarding TCs, FRITSCHE ANDREAS from the University of Tübingen, Germany, holds the first position and has the highest H-index. He mainly assesses the visceral adipose mass MRI and their correlation with markers for insulin resistance and prediabetes (19). It is crucial to consider the number of publications and the quality of their articles to identify prolific authors.

The annual publications output in the field of fMRI and diabetes mellitus demonstrated a continuous and stable upward trend in the past 30 years. Regarding countries/regions, China and the USA are the leading contributors to fMRI and diabetes mellitus research. Although China ranks first in the number of publications, it has a low position in TCs, indicating a lower quality of articles. The USA ranks first in TCs. The most cited article from the US was published in the *Proceedings of the National Academy of Sciences of the United States of America* in 2001, with 1986 citations (30), indicating a higher quality of articles. In addition, seven institutions among the top 10 are from the USA. In other words, the USA is significant in the field of fMRI and diabetes mellitus. In the USA, 30.3 million (9.4%) adults had diabetes mellitus in 2017 (31). The country has a good foundation in medical research and provides significant financial support for research. Overall, with irreversible globalization, there is still a need for more collaboration between countries/institutions to promote research in this field.

### 4.2 Research hotspots and trends

Among the top 20 most frequent keywords, the top five keywords were “dementia,” “risk,” “brain,” “Alzheimer’s disease,” and “functional connectivity.” In addition, the top 20 most cited keywords, listed in Figure 8, reveal the potential hotspots of fMRI and diabetes research in the past 30 years. Firstly, the “risk,” “dysfunction,” “impairment,” and “dementia” keywords suggest that the current literature mainly focuses on diabetes mellitus and the risk of cognitive impairment/dysfunction. Increasing studies suggest that T1DM and T2DM are associated with decreased performance in multiple domains of cognitive function and structural and fMRI abnormalities of the brain (27, 32, 33). Therefore, some researchers began to establish dementia risk prediction modeling with fMRI data to predict cognitive impairment/dysfunction and dementia. For example, Samoilova et al. used MRI data to create a computer neural network model for predicting the development of cognitive impairment in DM based on brain neuroimaging techniques (34). There are few studies on the prediction of cognitive function in patients with T2DM based on fMRI (35). Therefore, future research should present a new solution idea, which extracts the characteristics from fMRI and establishes a cognitive function prediction model among diabetes mellitus patients.

Secondly, the “dysfunction,” “impairment,” and “diagnosis” keywords indicated that one of the research hotspots is to find potential biomarkers for early diagnosis of cognitive impairment/dysfunction. Diabetes mellitus is associated with cognitive decline and altered brain structure (32). FC can be used to reflect brain function, diagnose neurodegenerative diseases, and provide insights into pathophysiologic mechanisms (36, 37). Blood oxygen level-dependent (BOLD) signaling in fMRI reveals hemodynamic changes associated with neural activity and has been used to detect FC alterations in patients with T2DM and mild cognitive impairment (MCI) (38–40). Region-specific FC provides useful features for T2DM-MCI diagnosis. Hence, FC is a potential biomarker for assessing the degree of cognitive decline (41). In addition, the application of multiple resting-state fMRI (rs-MRI) indices can detect abnormal neural activities in different brain regions in patients with T2DM, and a decreased coupling trend between the amplitude of low-frequency fluctuation (ALFF) can help understand the early changes before cognitive impairment appears. Compared with other fMRI techniques, rs-fMRI is widely used because it requires the least number of patients, acquires signals more easily, and is proficient in recognizing functional areas in many different groups, even those with low cooperation. Therefore, rs-fMRI may be useful for detecting central nervous system impairment caused by T2DM with DPN (14). Many studies explored the fMRI imaging of the brain in T2DM cognitive dysfunction (42–44). Research found that hippocampal atrophy can be used as an early imaging marker for structural changes in brain regions of patients with T2DM cognitive impairment (45). These findings provide some new insights into the neural mechanisms of diabetes mellitus-related cognitive impairment.

### 4.3 Strengths and limitations

This study is the first bibliometric analysis on fMRI and diabetes mellitus over the past 30 years, demonstrating a systematic overview of the field and providing guidance for future research. With the help of the present bibliometric analysis, researchers interested in fMRI and diabetes mellitus can get an overview of the field and quickly get up to speed on the latest research hotspots. However, there were some limitations to this study. Firstly, this study’s reliance on specific databases may have excluded relevant literature not indexed in the SCI-E database from WoSCC. Secondly, while bibliometric methods provide valuable insights, they have limitations in capturing the full scope and nuances of the research landscape. Finally, the generalizability of findings may be limited to the specific context of fMRI in diabetes mellitus research.

## 5 Conclusion

The present study analyzed the research on fMRI and diabetes mellitus in the past 30 years with the help of bibliometric mapping. The results suggest that the overall publication numbers maintained a stable growth trend. China and the USA published the largest number of papers, highlighting their extensive research contribution in this field. Among the top ten institutions based on the number of publications, the majority of articles are from the USA. Diabetic cognitive impairment/dysfunction and diabetic brain activity are the most intensive research topics. Future research should focus more on predicting cognitive function in patients with diabetes mellitus based on fMRI.

## Data Availability

The original contributions presented in the study are included in the article/supplementary material, further inquiries can be directed to the corresponding authors.

## Footnotes

^1^ https://bibliometric.com/
